# Functional Analysis of the Heat-Tolerance Candidate Gene *BcNCED3* in Non-Heading Chinese Cabbage (*Brassica campestris* ssp. *chinensis*)

**DOI:** 10.3390/plants15172613

**Published:** 2026-08-27

**Authors:** Wenjun Lu, Changwei Zhang, Zhaokun Liu, Chenyu Chen, Yaolong Wang, Sheng Shu, Hongfang Zhu, Dong Xiao

**Affiliations:** 1 Sanya Institute of Nanjing Agricultural University, Sanya 572024, China; 2 National Key Laboratory of Crop Genetics & Germplasm Enhancement and Utilization, Nanjing Agricultural University, Nanjing 210095, China; changweizh@njau.edu.cn (C.Z.);; 3 Vegetable Research Institute, Suzhou Academy of Agricultural Sciences, Suzhou 215155, China; 4 Shanghai Key Laboratory of Facility Horticulture Technology, Shanghai Academy of Agricultural Sciences, Shanghai 200062, China

**Keywords:** non-heading Chinese cabbage, heat stress, *BcNCED3*, abscisic acid, CRISPR/Cas12a

## Abstract

Non-heading Chinese cabbage (*Brassica campestris* ssp. *chinensis*) is an economically important leafy vegetable whose quality and yield are severely constrained by high temperature. Abscisic acid (ABA) is a key stress hormone, and 9-*cis*-epoxycarotenoid dioxygenase (*NCED*) is the rate-limiting enzyme of its biosynthesis; however, the function of *NCED* in the thermotolerance of this crop remains unclear. In this study, the heat-tolerance candidate gene *BcNCED3* was functionally characterized in non-heading Chinese cabbage using bioinformatics, subcellular localization, expression profiling, exogenous ABA treatment, genetic transformation, and CRISPR/Cas12a editing. *BcNCED3* has an intronless 1797 bp open reading frame encoding a 598-amino-acid hydrophilic protein with a conserved RPE65 domain, is localized to the chloroplast, and is highly conserved across *Brassicaceae*. Its transcript was strongly induced under 40 °C heat stress, with reaching markedly higher transcript abundance at 6 h in the heat-tolerant cultivar ‘Suzhou Qing’ than in the heat-sensitive ‘Aijiaohuang’. Exogenous ABA (10 µM) pretreatment alleviated heat injury, increased endogenous ABA content by 1.8-fold, upregulated heat-responsive genes (*BcHSFA1*, *BcHSFA2*, *BcHSP101*, *BcDREB2A*, *BcMBF1c* and *BcGolS1*) by 1.5- to 3.2-fold, enhanced POD, SOD, and CAT activities by 25–40%, and reduced malondialdehyde accumulation by 32%. *BcNCED3*-overexpressing plants with 12- to 30-fold increased expression reproduced these responses, whereas Cas12a knockout plants exhibited heat hypersensitivity. These results demonstrate that *BcNCED3* positively regulates thermotolerance in non-heading Chinese cabbage and provide a genetic resource for heat-tolerance breeding.

## 1. Introduction

Non-heading Chinese cabbage (*Brassica campestris* ssp. *chinensis*) originated in China and is an important leafy vegetable of the family *Brassicaceae* with high nutritional and economic value [1]. It is a cool-season crop that grows best at temperatures between 18 and 25 °C. Sustained high temperatures inhibit growth, causing dwarfism, leaf yellowing, reduced nutritional quality, lower survival, and increased disease susceptibility, thereby severely affecting the yield and quality. Since industrialization, the global mean temperature has risen by approximately 1.1 °C, and the frequency, intensity, and duration of heat waves are increasing rapidly [2]. A 1 °C rise in temperature is estimated to reduce the yield of major crops by 3–7% [3,4], posing a serious threat to the year-round stable supply of this crop. Therefore, elucidating the molecular mechanisms underlying heat stress responses in non-heading Chinese cabbage and developing heat-tolerant cultivars have become urgent priorities.

Plants respond to heat stress through a highly coordinated molecular network. Heat stress transcription factors (HSFs) and heat shock proteins (HSPs) serve as molecular chaperones to maintain protein homeostasis [5,6,7]. Concurrently, reactive oxygen species (ROS) generated under thermal stress are detoxified by antioxidant enzymes, including superoxide dismutase (SOD), peroxidase (POD), and catalase (CAT) [8,9,10]. Phytohormones, particularly abscisic acid (ABA), provide central regulatory oversight, integrating these protective pathways [11]. ABA, widely regarded as the principal stress hormone, accumulates rapidly under drought, salinity, and high-temperature conditions [12].

In plants, ABA is synthesized via the carotenoid (indirect) pathway [13]. In plastids, *β*-carotene is converted to zeaxanthin and then to all-trans-violaxanthin, which is cleaved by 9-cis-epoxycarotenoid dioxygenase (NCED) to form the C15 precursor xanthoxin, which is subsequently oxidized in the cytosol to yield ABA [14,15]. *NCED* catalyzes the committed rate-limiting step, and its expression directly determines the rate of ABA accumulation [16]. Upon heat perception, ABA is recognized by *PYR*/*PYL*/*RCAR* receptors, which inhibit type 2C protein phosphatases (PP2Cs), thereby activating *SnRK2* kinases and downstream *AREB*/*ABF* transcription factors to activate stress-responsive genes [17,18]. ABA acts as a central regulator of plant thermotolerance [19]. Under heat stress, it promotes stomatal closure to limit water loss and acts as a signal that activates heat-responsive genes [20]. In tomato, *SlSnRK2*.3 modulates ABA-controlled stomatal movement and confers high-temperature tolerance [21]; in wheat, the heat-induced *TaABF5b*–*HsfA2h*/*HsfC2a* module directly activates *TaNCED2b* to promote ABA synthesis and enhance thermotolerance [22]; and in maize, *ZmbZIP4* improves seedling heat tolerance by promoting ABA biosynthesis [23]. Exogenous ABA applied before heat stress can enhance sucrose metabolism, antioxidant capacity, and heat shock gene expression [24,25].

*NCED* genes typically constitute small multigene families characterized by conserved RPE65 domains, with individual members exhibiting distinct tissue- and stress-specific expression patterns [26,27]. Since the founding member VP14 was cloned from the maize *vp14* mutant [28,29], *NCED* genes have been identified across diverse plant species [30,31,32] and characterized at the genome-wide level in crops such as soybean and wheat [33,34]. Functionally, *NCED* family members regulate seed dormancy and germination, root and floral development, and fruit ripening through spatiotemporal control of ABA biosynthesis [35,36,37,38,39]. They play central roles in abiotic stress tolerance, conferring drought, salt, and cold resistance in *Arabidopsis*, rice, and peanut [40,41,42,43,44,45,46,47].

Several heat tolerance genes have been reported in non-heading Chinese cabbage, including *BchProT1* [48], *BcWRKY22* [49], and the *BcWRKY23*/*BcWRKY25* module [50]. Although *NCED* is a well-established rate-limiting enzyme in ABA biosynthesis, its function in this crop remains largely unexplored. In this study, we selected *BcNCED3-*a heat tolerance candidate gene identified through prior resequencing and genetic mapping for functional characterization in non-heading Chinese cabbage. We systematically analyzed its gene and protein architecture, subcellular localization, and heat-inducible expression dynamics. Its biological function was validated using complementary approaches including exogenous ABA supplementation, constitutive overexpression, and CRISPR/Cas12a-mediated targeted mutagenesis. This work provides both mechanistic insights and genetic resources to advance heat-tolerance breeding in non-heading Chinese cabbage.

## 2. Materials and Methods

### 2.1. Plant Materials and Growth Conditions

‘Suzhou Qing’ (NHCC001) is a locally adapted landrace from Jiangsu Province with documented heat tolerance, while ‘Aijiaohuang’ (NHCC002) is a commercial F_1_ hybrid known to be heat-sensitive. Both cultivars are routinely maintained by the Cabbage Systems Biology Laboratory at Nanjing Agricultural University. Their contrasting thermotolerance phenotypes have been validated through multi-year field observations and controlled-environment assays. Seeds were germinated on moist filter paper, sown into 50-cell trays containing substrate (nutrient soil:vermiculite = 2:1), and uniform two-leaf-one-heart seedlings were transplanted into 6.5 cm pots. All plants were grown in a light incubator (24 °C/18 °C, 16 h light/8 h dark, relative humidity 60 ± 5%).

### 2.2. RNA Extraction and cDNA Synthesis

Fresh leaves (0.1 g) were frozen in liquid nitrogen and ground into a fine powder. Total RNA was extracted using the RNAsimple Total RNA Kit (TIANGEN Biotech (Beijing) Co., Ltd., Beijing, China) following the manufacturer’s instructions, and RNA quality was assessed by ultraviolet spectrophotometry (A260/A280 = 1.8–2.1). First-strand cDNA was synthesized using the Evo M-MLV reverse-transcription premix kit (Accurate Biotechnology (Hunan) Co., Ltd., Changsha, China) and stored at −20 °C for cloning, vector construction, and quantitative PCR.

### 2.3. Bioinformatic Analysis of BcNCED3

The *AtNCED3* (AT3G14440) sequence was retrieved from TAIR (https://www.arabidopsis.org/, accessed on 5 November 2024), and the *BcNCED3* gene, coding sequence (CDS), and protein sequence were obtained from the published non-heading Chinese cabbage genome [51] using homology comparison in TBtools (v2.056). The physicochemical properties and hydrophobicity were analyzed using ProtParam and ProtScale (ExPASy) (Swiss Institute of Bioinformatics, Geneva, Switzerland), conserved domains using SMART (European Molecular Biology Laboratory (EMBL), Heidelberg, Germany), transmembrane structure using DeepTMHMM (Technical University of Denmark, Kgs Lyngby, Denmark), secondary and tertiary structures using SOPMA (Institut de Biologie et de Chimie des Protéines (IBCP‑CNRS), Lyon, France) and SWISS-MODEL (Swiss Institute of Bioinformatics & University of Basel, Basel), respectively, and subcellular localization was predicted using CELLO v2.5 (National Chiao Tung University, Hsinchu, Taiwan, China) and Cell-PLoc (Gordon Life Science Institute, San Diego, CA, USA; Shanghai Jiao Tong University, Shanghai, China).

### 2.4. Promoter Cis-Acting Element Analysis

The 2000 bp sequence upstream of the *BcNCED3* start codon (ATG) was downloaded from NCBI, and *cis*-acting elements were predicted using PlantCARE and visualized in TBtools.

### 2.5. Sequence Alignment and Phylogenetic Analysis

The BcNCED3 protein sequence was used as a query against the BRAD database (http://brassicadb.cn/) using BLAST (V 4.0). Homologous sequences from species with ≥80% identity were aligned using DNAMAN6, and a phylogenetic tree was constructed in MEGA12 using the neighbor-joining method with 1000 bootstrap replicates [52].

### 2.6. Gene Cloning

Specific primers were designed using Primer-BLAST (Table 1). Target fragments were amplified from cDNA using PrimeSTAR Max DNA Polymerase (Takara Bio Inc., Kusatsu, Shiga, Japan), separated on 1.2% agarose gels, recovered using a gel-extraction kit (TIANGEN Biotech (Beijing) Co., Ltd., Beijing, China), and stored at −20 °C.

### 2.7. Subcellular Localization

The stop codon was removed, and the *BcNCED3* CDS was inserted into the *Nde* I/*Bam* H I-digested pRI101-EGFP vector by homologous recombination (OK Clon DNA Ligation Kit III, Accurate Biotechnology (Hunan) Co., Ltd., Changsha, China) to generate 35S:*BcNCED3*-GFP. The recombinant and empty (35S:GFP) vectors were transformed into *Agrobacterium tumefaciens* GV3101 (pSoup-p19) and infiltrated into the abaxial epidermis of four-week-old *N. benthamiana* leaves. After 24 h of darkness followed by 1–2 d of normal light, GFP and chloroplast autofluorescence were observed using a confocal laser-scanning microscope (Zeiss LSM780, Carl Zeiss Microscopy GmbH, Jena, Germany).

### 2.8. High-Temperature Treatment and Expression Profiling

Four-week-old ‘Suzhou Qing’ and ‘Aijiaohuang’ plants were exposed to 40 °C, and the third true leaf was sampled at 0, 3, 6, 9, and 12 h (three plants per time point, three biological replicates). Samples were frozen in liquid nitrogen and used for RNA extraction and RT-qPCR, as described in Section 2.2.

### 2.9. Real-Time Quantitative PCR (RT-qPCR)

Gene-specific primers (Table 1) were used with AceQ Universal SYBR qPCR Master Mix (Vazyme Biotech Co., Ltd., Nanjing, China) on a Bio-Rad CFX96 system (two-step amplification: 95 °C for 5 min; 40 cycles of 95 °C for 10 s and 60 °C for 30 s, followed by melt-curve analysis). *BcACTIN7* served as the internal reference, and relative expression was calculated using the 2^−ΔΔCt^ method with three technical replicates [53].

### 2.10. Exogenous ABA Treatment and Heat Stress

Four-week-old ‘Aijiaohuang’ seedlings were sprayed with 10 µmol·L^−1^ ABA on both leaf surfaces once daily for three days before heat stress, whereas controls received distilled water. Plants were then exposed to 38 °C/25 °C (16 h light/8 h dark, RH 60 ± 5%) for three days with equal daily watering to exclude drought effects. The third fully expanded functional leaf was sampled before and after treatment, frozen in liquid nitrogen, and stored at −80 °C. Transgenic and non-transgenic plants were heat-treated under identical conditions.

### 2.11. Overexpression Vector Construction and Genetic Transformation

The *BcNCED3* CDS (stop codon removed) was inserted into the *Nru* I site of the pER8-WPR-superGFP vector by homologous recombination and transformed into *Agrobacterium rhizogenes* K599. Seeds were surface-sterilized and germinated on 1/2 MS medium. Explants comprising two cotyledons and 1 cm hypocotyl were infiltrated with *A. rhizogenes* (OD600 0.6–0.8, 150 µmol·L^−1^ acetosyringone), co-cultured in darkness for 2d, and transferred through differentiation, subculture, and rooting media. Roots showing green GFP fluorescence under a handheld lamp (LUYOR-3415RG) were selected, and transgenic plants were confirmed by PCR and RT-qPCR. The transformation system followed the *A. rhizogenes*-mediated method reported for Chinese cabbage [54], with some modifications.

### 2.12. CRISPR/Cas12a Vector Construction and Editing

The 35S:LbCas12a-RRV expression cassette was cloned into the WUS2-IPT-PLT5-Ruby backbone to construct the LbCas12a-RRV vector [55]. crRNAs targeting the *BcNCED3* open reading frames were designed using CRISPR-P v2.0 (Table 2). *BcNCED3*-edited regenerants were genotyped by PCR and third-generation sequencing.

### 2.13. Determination of ABA Content and Physiological Indices

Endogenous ABA content was quantified using a plant ABA ELISA kit (Shanghai Enzyme-linked Biotechnology Co., Ltd., Shanghai, China; Catalog No. ml064291) at 450 nm against a standard curve, following the manufacturer’s protocol. Absorbance values were interpolated from a six-point ABA standard curve (0–100 ng·mL^−1^). Malondialdehyde (MDA) concentration was determined by the thiobarbituric acid (TBA) colorimetric method at 532 nm. Catalase (CAT), total superoxide dismutase (T-SOD), and peroxidase (POD) activities were measured using commercial assay kits (Nanjing Jiancheng Bioengineering Institute, Nanjing, China/Geruisi Biotechnology, Suzhou, China) at 510, 550, and 420 nm, respectively. Statistical analyses were performed as follows: pairwise comparisons (e.g., ABA-treated vs. control gene expression) used Student’s t-test; multi-group comparisons of gene expression in overexpression and genome-edited lines employed one-way ANOVA followed by Dunnett’s post-hoc test; all physiological index comparisons across genotypes and treatments applied two-way ANOVA followed by Tukey’s honestly significant difference (HSD) test. All analyses were conducted in SPSS 26.0 (IBM Corp., Armonk, NY, USA), and graphs were generated using GraphPad Prism 8 (three biological replicates per treatment).

## 3. Results

### 3.1. Gene and Protein Structural Features of BcNCED3

**Figure 1:**
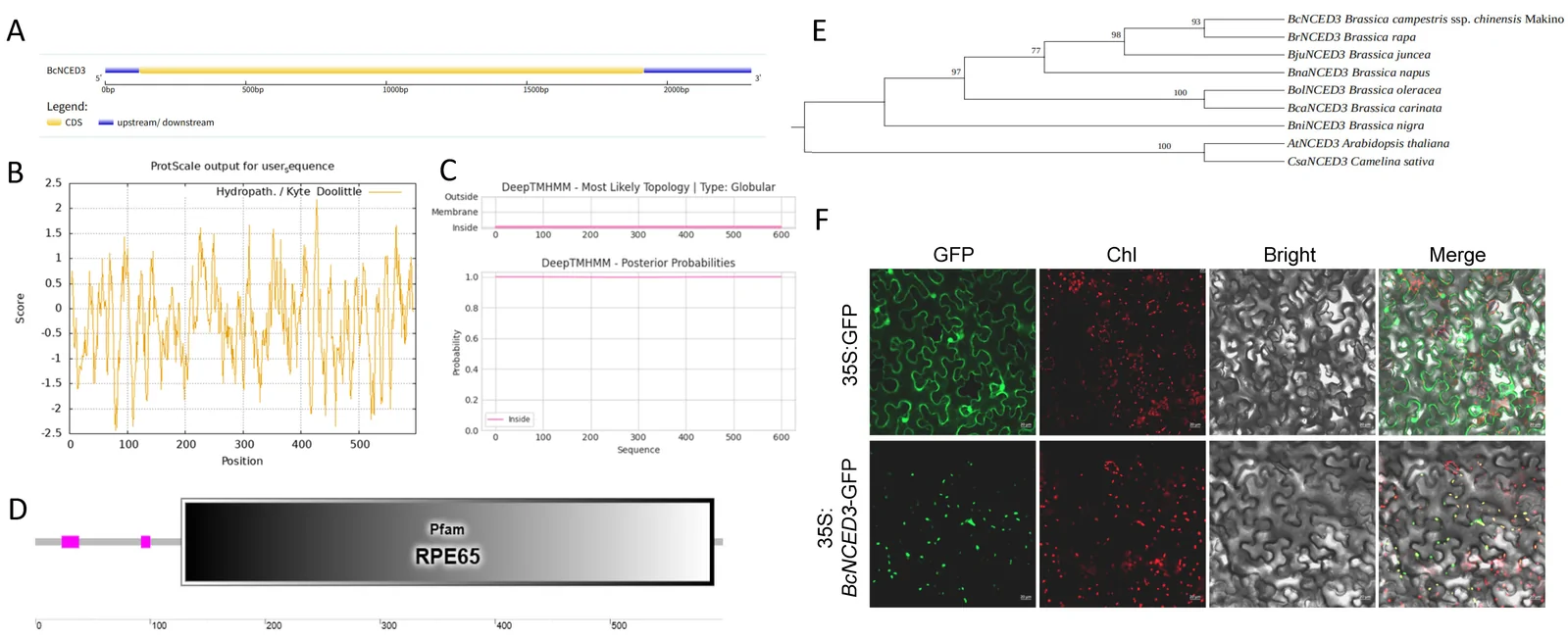
Bioinformatic analysis and subcellular localization of *BcNCED3*: (**A**) gene structure showing CDS and flanking regions; (**B**) hydrophobicity analysis of *BcNCED3*; (**C**) transmembrane topology prediction; (**D**) conserved RPE65 domains; (**E**) phylogenetic tree of NCED3 proteins from eight *Brassicaceae species* (*Brassica rapa*, *B. juncea*, *B. napus*, *B. oleracea*, *B. carinata*, *B. nigra*, *Arabidopsis thaliana*, and *Camelina sativa*), constructed using the neighbor-joining method with 1000 bootstrap replicates (bootstrap values (≥70%) are shown at branch nodes, indicating the statistical confidence of each bifurcation); (**F**) subcellular localization of *BcNCED3* in *Nicotiana benthamiana* epidermal cells: 35S:GFP control and 35S:*BcNCED3*-GFP showing GFP, chloroplast autofluorescence (Chl), bright field (Bright), and merged channels.

The full-length gene *BcNCED3* (BraC01g041920.1) spans 2299 bp, harboring a single 1797 bp coding sequence devoid of intronic regions. This locus encodes a 598-amino-acid polypeptide (Figure 1A). Biophysical parameter calculation predicted a molecular weight of 65.76 kDa and a theoretical pI of 5.57, accompanied by an instability index of 41.45. Global hydrophilicity profiling indicated that the protein is predominantly soluble (Figure 1B), with no predicted transmembrane helices detected (Figure 1C), collectively supporting that *BcNCED3* constitutes an acidic, labile soluble protein.

In silico secondary structure prediction uncovered three dominant structural components: random coils occupied the largest proportion at 66.39%, followed by extended strands (20.40%) and α-helices (13.21%). Homology-based tertiary structure modeling was performed using the *Arabidopsis NCED3* template, sharing 91.47% sequence identity with *BcNCED3*; the resulting 3D conformation matched the secondary structural distribution. The polypeptide carries a canonical RPE65 conserved domain spanning residues 127 to 529 (Figure 1D). Subcellular localization prediction assigned *BcNCED3* to chloroplast compartments, consistent with its core catalytic role in plastid-localized ABA biosynthetic cascades.

### 3.2. Cis-Regulatory Motifs Within the BcNCED3 Promoter

Beyond basal core promoter elements, the 2000 bp upstream regulatory region of *BcNCED3* accumulates abundant *cis*-elements responsive to phytohormones and environmental stimuli. Identified hormone-associated motifs include ABA-responsive ABRE, methyl jasmonate-inducible CGTCA-motif and ethylene-triggered ERE. Stress-related signatures were also captured, such as anaerobic induction ARE and general stress-responsive STRE motifs. Multiple binding cis-sites for transcription factors *MYB*, *MYC* and *WRKY* (W-box), alongside a suite of light-responsive modules, were additionally distributed across the promoter fragment (Supplementary Materials).

Pervasive ABRE enrichment points to tight transcriptional control mediated by ABA signaling cascades. The coexistence of STRE motifs further implies that *BcNCED3* acts as a molecular hub integrating ABA and thermal signals to coordinate plant thermotolerance.

### 3.3. Multiple Sequence Alignment and Phylogenetic Reconstruction

Homology searches against the BRAD database retrieved eight *Brassicaceae* species whose *NCED3* orthologs shared over 80% amino acid identity with *BcNCED3*, encompassing *Brassica rapa*, *B. juncea*, *B. napus*, *B. oleracea*, *B. carinata*, *B. nigra*, *Arabidopsis thaliana* and *Camelina sativa*. Pairwise identity values between *BcNCED3* and these homologous proteins reached 100%, 99.8%, 98.7%, 98.5%, 98%, 96%, 91.5% and 92%, respectively.

Intra-genus sequence homology within *Brassica* consistently surpassed 98%, while cross-genus similarity remained above 90%, reflecting profound functional conservation of *NCED3* across cruciferous taxa. The reconstructed phylogenetic tree partitioned all *Brassica* orthologs into a monophyletic clade, separating *Arabidopsis* and *Camelina* into an independent branch (Figure 1E), substantifying the deep evolutionary conservation of *NCED3* within *Brassicaceae*.

### 3.4. Experimental Validation of BcNCED3 Subcellular Localization

This assay was designed to validate *BcNCED3’s* contribution to plastid-resident ABA biosynthesis and furnish cytological evidence supporting its stress-modulatory functions in non-heading Chinese cabbage. Although chloroplast localization of *NCED* homologs has been established in model species such as *Arabidopsis thaliana* and tomato, divergent transit peptide sequences among *NCED* orthologs in horticultural crops may drive distinct intracellular sorting patterns. We therefore experimentally validated the subcellular distribution of *BcNCED3* through transient expression of a 35S:*BcNCED3*-GFP fusion construct in *Nicotiana benthamiana* leaf epidermal cells-a well-established heterologous platform for cytological characterization of vegetable crop genes. The 35S:GFP empty vector control yielded diffuse fluorescence throughout the cytosol and nucleus, showing no overlap with chloroplast autofluorescence. In contrast, the *BcNCED3*-GFP fusion protein produced discrete punctate signals that fully colocalized with chloroplast autofluorescence, yielding characteristic yellow signals in merged micrographs (Figure 1F). These cytological observations provide direct experimental evidence for chloroplast targeting of *BcNCED3*, confirming its catalytic function within the plastid-localized ABA biosynthetic pathway in non-heading Chinese cabbage.

### 3.5. Heat-Triggered Transcriptional Profiling of BcNCED3

**Figure 2:**
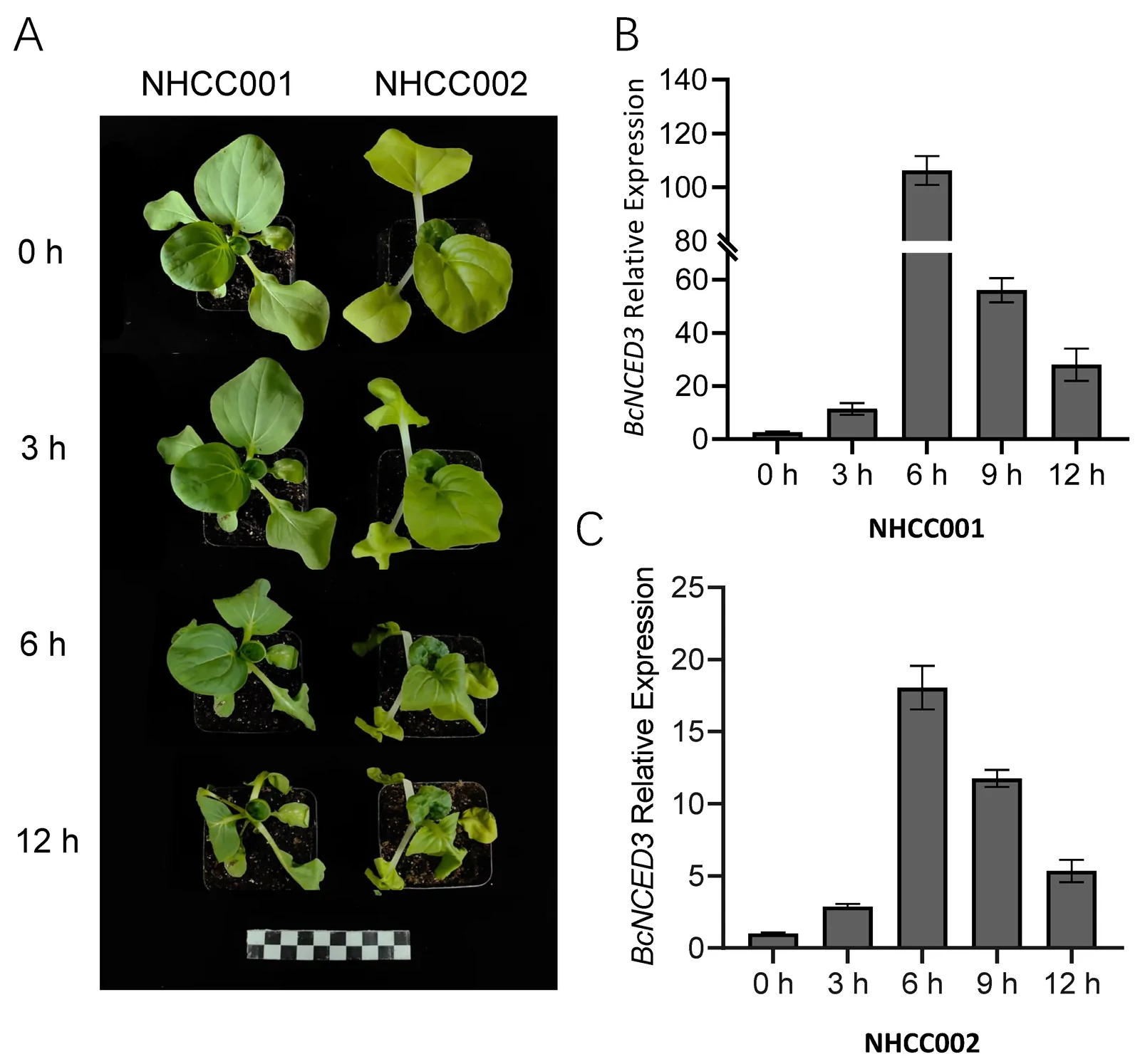
Phenotypic changes and relative expression of *BcNCED3* in NHCC001 and NHCC002 under high temperature: (**A**) phenotypes of NHCC001 and NHCC002 at 0, 3, 6, and 12 h of 40 °C treatment; (**B**,**C**) relative expression of *BcNCED3* in NHCC001 (**B**) and NHCC002 (**C**). Data are presented as mean ± SD (*n* = 3 biological replicates). Asterisks denote statistically significant differences (Student’s *t*-test).

Quantitative RT–PCR was employed to quantify *BcNCED3* transcript abundance over a 0–12 h 40 °C heat stress regime. Two non-heading Chinese cabbage genotypes with divergent thermotolerance were assayed: thermotolerant cultivar ‘Suzhou Qing’ (NHCC001) and heat-vulnerable ‘Aijiaohuang’ (NHCC002). Heat-triggered leaf wilting was markedly exacerbated in the heat-sensitive line (Figure 2A). *BcNCED3* transcripts accumulated to higher levels in NHCC001 throughout the stress period, and heat exposure triggered robust transcriptional upregulation in both genotypes. Transcript quantities rose sharply from 3 h to 6 h post-treatment, peaking at a roughly 5-fold induction at 6 h before undergoing a pronounced reduction by 9 h of thermal exposure (Figure 2B,C). These transcriptional patterns establish *BcNCED3* as a heat-inducible gene, whose activation amplitude aligns positively with intrinsic thermotolerance capacity of non-heading Chinese cabbage cultivars.

### 3.6. Exogenous ABA Pretreatment Fortifies Basal Thermoprotection

**Figure 3:**
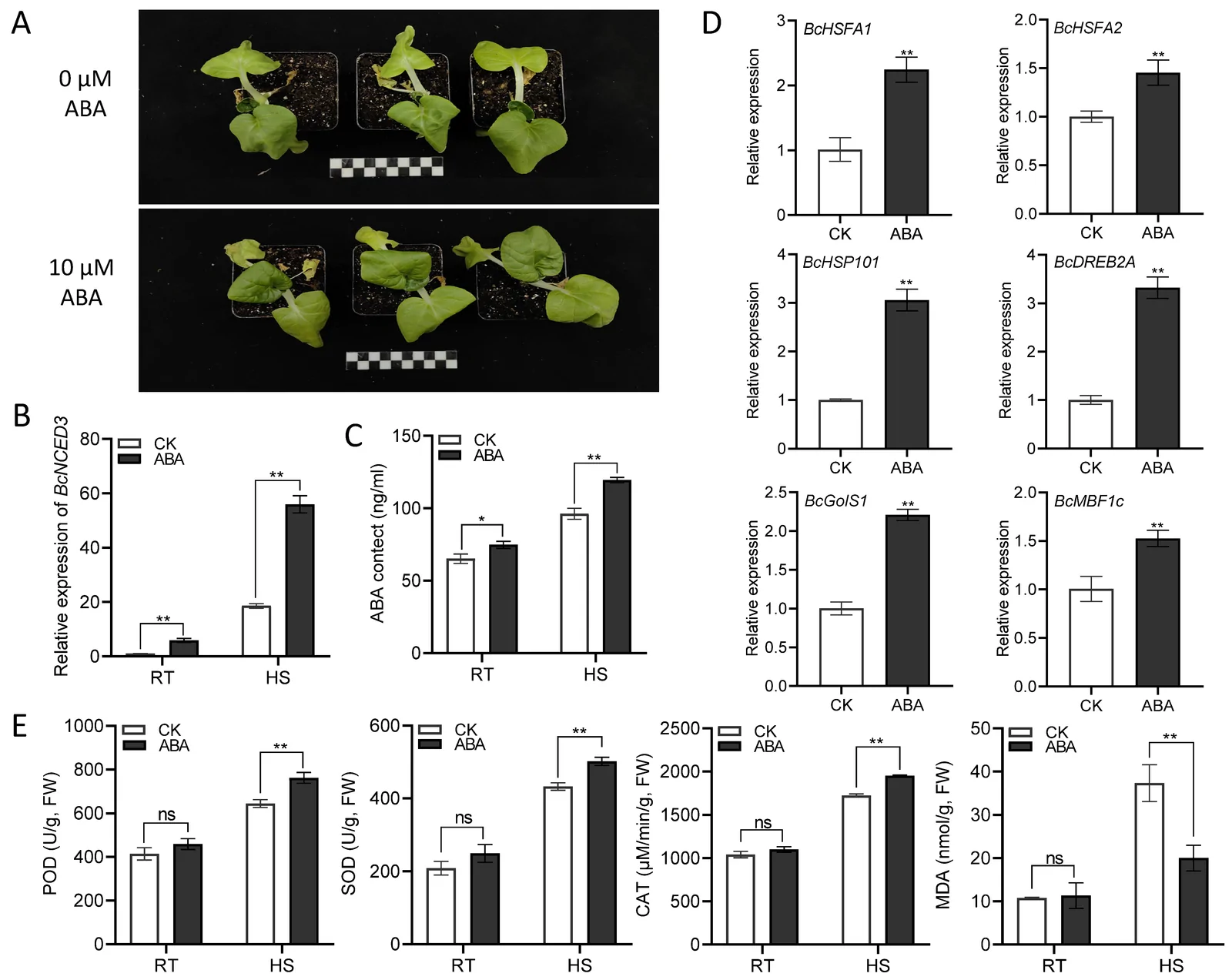
Effects of exogenous ABA pretreatment on heat tolerance of non-heading Chinese cabbage: (**A**) phenotypes of the control (CK) and ABA-pretreated (ABA) groups after high-temperature treatment; (**B**) relative expression of *BcNCED3* in the CK and ABA groups at room temperature (RT, 24 °C) and high temperature (HS, 38 °C); (**C**) endogenous ABA levels; (**D**) expression of heat-responsive genes (*BcHSFA1*, *BcHSFA2*, *BcHSP101*, *BcDREB2A*, *BcGolS1*, and *BcMBF1c*) after high-temperature treatment; (**E**) POD, SOD, and CAT activities and MDA content at RT and HS. Data are presented as mean ± SD (*n* = 3 biological replicates). Asterisks indicate statistically significant differences (* *p* < 0.05, ** *p* < 0.01, Student’s *t*-test for gene expression; two-way ANOVA with Tukey’s HSD for physiological indices, ns = non-significant).

Seedlings of heat-sensitive ‘Aijiaohuang’ primed with 10 µM ABA exhibited attenuated heat lesions following three days of cycling 38 °C/25 °C conditions, compared with water-mock controls. Untreated control material developed extensive chlorosis and necrotic lesions on basal leaves coupled with severe apical wilting, whereas ABA-pretreated foliage retained superior stress resilience (Figure 3A). Both *BcNCED3* transcript abundance and intrinsic ABA pools accumulated to higher titres in ABA-primed seedlings relative to mock specimens, with further amplification recorded upon heat imposition (Figure 3B,C). These observations establish that exogenous ABA signalling drives transcriptional activation of *BcNCED3* and stimulates de novo endogenous ABA biosynthesis.

Transcripts encoding core heat-responsive regulators-*BcHSP101*, *BcHSFA1*, *BcHSFA2*, *BcDREB2A*, *BcGolS1,* and *BcMBF1c*-were significantly upregulated in ABA-pretreated seedlings post heat exposure (Figure 3D). This transcriptional cascade infers that ABA-activated *BcNCED3* signalling reinforces thermotolerance by mobilizing downstream heat shock regulons.

Basal antioxidant enzyme activities and malondialdehyde (MDA) titres were indistinguishable across treatment cohorts prior to thermal stress. After three days of high-temperature cycling, ABA priming yielded substantially elevated SOD, POD and CAT enzymatic activities, alongside restrained MDA accumulation relative to untreated controls (Figure 3E). Exogenous ABA therefore reinforces cellular antioxidant buffering capacity, accelerates reactive oxygen species (ROS) scavenging and curtails lipid peroxidation, thereby preserving plasma membrane integrity under thermal duress.

### 3.7. Constitutive Overexpression of BcNCED3 Enhances Intrinsic Thermotolerance

We generated regenerated ‘Aijiaohuang’ seedlings by *Agrobacterium*-mediated transformation and subsequent plant tissue regeneration culture. We irradiated seedling roots with the LUYOR-3415RG handheld fluorescent lamp, and strong green GFP fluorescence signals were clearly visible in positive transgenic individuals when viewed through a yellow filter. Tissue culture successfully yielded transgenic ‘Aijiaohuang’ regenerants with GFP reporter signals specifically expressed in roots (Supplementary Figure S2A). Genotypic screening using primer pairs pER8-BcNCED3-F/pER8-R identified three independent lines carrying the expected ~2000 bp amplicon (Supplementary Figure S2B). Quantitative RT–PCR analysis revealed *BcNCED3* transcript levels in OE-1, OE-2, and OE-3 to reach approximately 30-, 12-, and 13-fold relative to wild-type (WT) expression, respectively (Supplementary Figure S2C). All three independent overexpression lines were retained for subsequent phenotypic and physiological analyses to demonstrate the consistency and reproducibility of the overexpression phenotype across independent transgenic events, thereby ruling out line-specific artifacts arising from positional effects or somaclonal variation.

**Figure 4:**
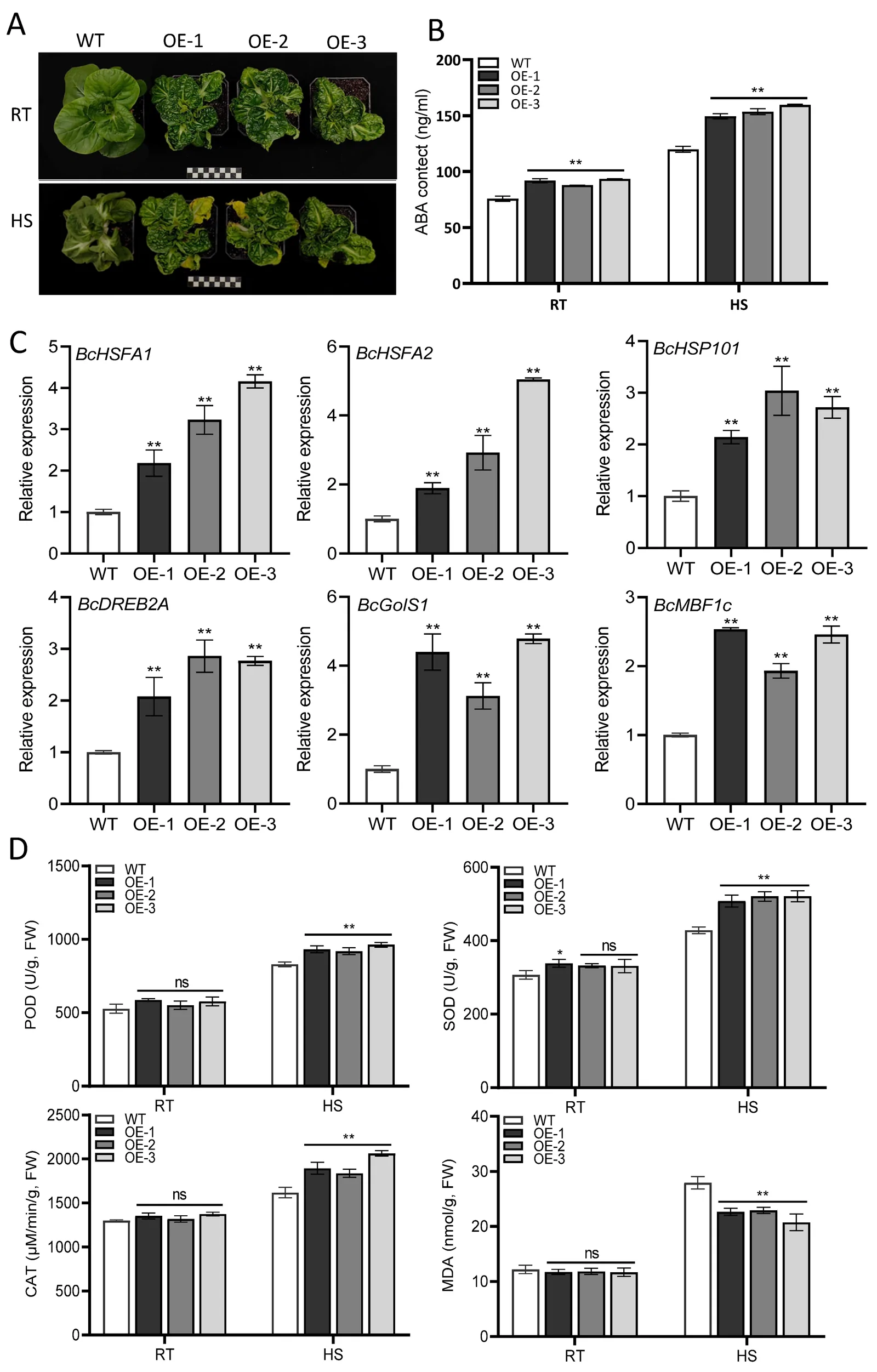
*BcNCED3* overexpression enhances heat tolerance in non-heading Chinese cabbage: (**A**) Phenotypes of WT and overexpression plants (OE-1, OE-2, and OE-3) at room temperature (RT, 24 °C) and high temperature (HS, 38 °C); (**B**) Endogenous ABA levels; (**C**) Expression of heat-responsive genes *BcHSFA1*, *BcHSFA2*, *BcHSP101*, *BcDREB2A*, *BcGolS1*, and *BcMBF1c* after high-temperature treatment; (**D**) POD, SOD, and CAT activities and MDA content at RT and HS. Data are presented as mean ± SD (*n* = 3 biological replicates), (* *p *< 0.05, ** *p *< 0.01, one-way ANOVA with Dunnett’s test for gene expression; two-way ANOVA with Tukey’s HSD for physiological indices, ns = non-significant).

Following three days of 38 °C/25 °C heat cycling, WT foliage displayed widespread chlorosis and wilting, while overexpression lines sustained intact newly emerged green tissue with only mild senescence in mature leaves (Figure 4A). Thermal stress stimulated endogenous ABA biosynthesis across all genotypes, yet overexpression lines maintained constitutively higher ABA concentrations under both ambient and heat-challenged regimes (Figure 4B). This demonstrates that ectopic *BcNCED3* expression mitigates heat-induced damage via sustained elevation of intrinsic ABA pools. All six characterized heat-responsive marker genes exhibited significantly stronger transcriptional activation in overexpression backgrounds upon heat imposition (Figure 4C). At the physiological level, stress-triggered increments in SOD, POD and CAT activity were amplified in *BcNCED3*-overexpressing lines, concurrent with suppressed MDA build-up compared with WT counterparts (Figure 4D). Such physiological readouts verify that constitutive *BcNCED3* expression reinforces antioxidant defence cascades and stabilizes membrane architecture during sustained heat exposure.

Pearson correlation analysis was used to assess the relationships between *BcNCED3* transcript abundance and the expression of six heat-responsive genes (*BcHSFA1*, *BcHSFA2*, *BcHSP101*, *BcDREB2A*, *BcGolS1*, and *BcMBF1c*), as well as the activities of SOD, POD and CAT antioxidant enzymes and MDA content under HS conditions. The full correlation matrix is provided in Supplementary Figure S3. Correlation analysis revealed that *BcNCED3* expression was significantly positively correlated with the transcript levels of all six heat-responsive genes, with correlation coefficients of 0.72 to 0.89 (*p* < 0.01). Moreover, *BcNCED3* transcript abundance was positively and significantly correlated with the activities of SOD (*r* = 0.81, *p* < 0.01), POD (*r* = 0.76, *p* < 0.01) and CAT (*r* = 0.69, *p* < 0.01). Conversely, *BcNCED3* expression exhibited a significant negative correlation with MDA content (*r* = −0.78, *p* < 0.01).

### 3.8. Loss-of-Function Mutagenesis of BcNCED3 Compromises Thermotolerance

Genomic DNA isolated from healthy regenerated plantlets was PCR-amplified using Cas12a vector-specific primers. Five independent transformants yielding the diagnostic ~800 bp amplicon were confirmed as transgene-positive. Long-read third-generation (PacBio) sequencing across both target loci resolved three distinct mutational classes, all involving small indel deletions within the *BcNCED3* coding sequence. The three knockout lines-designated KO-1, KO-2, and KO-3-harbored deletions ranging from 12 to 29 bp at the crRNA1 and crRNA2 target sites, resulting in frameshift mutations and premature stop codons. These mutations are predicted to yield truncated *BcNCED3* polypeptides lacking the catalytically essential RPE65 domain (Supplementary Figure S4A–C). All three homozygous knockout lines were advanced for thermotolerance phenotyping.

**Figure 5:**
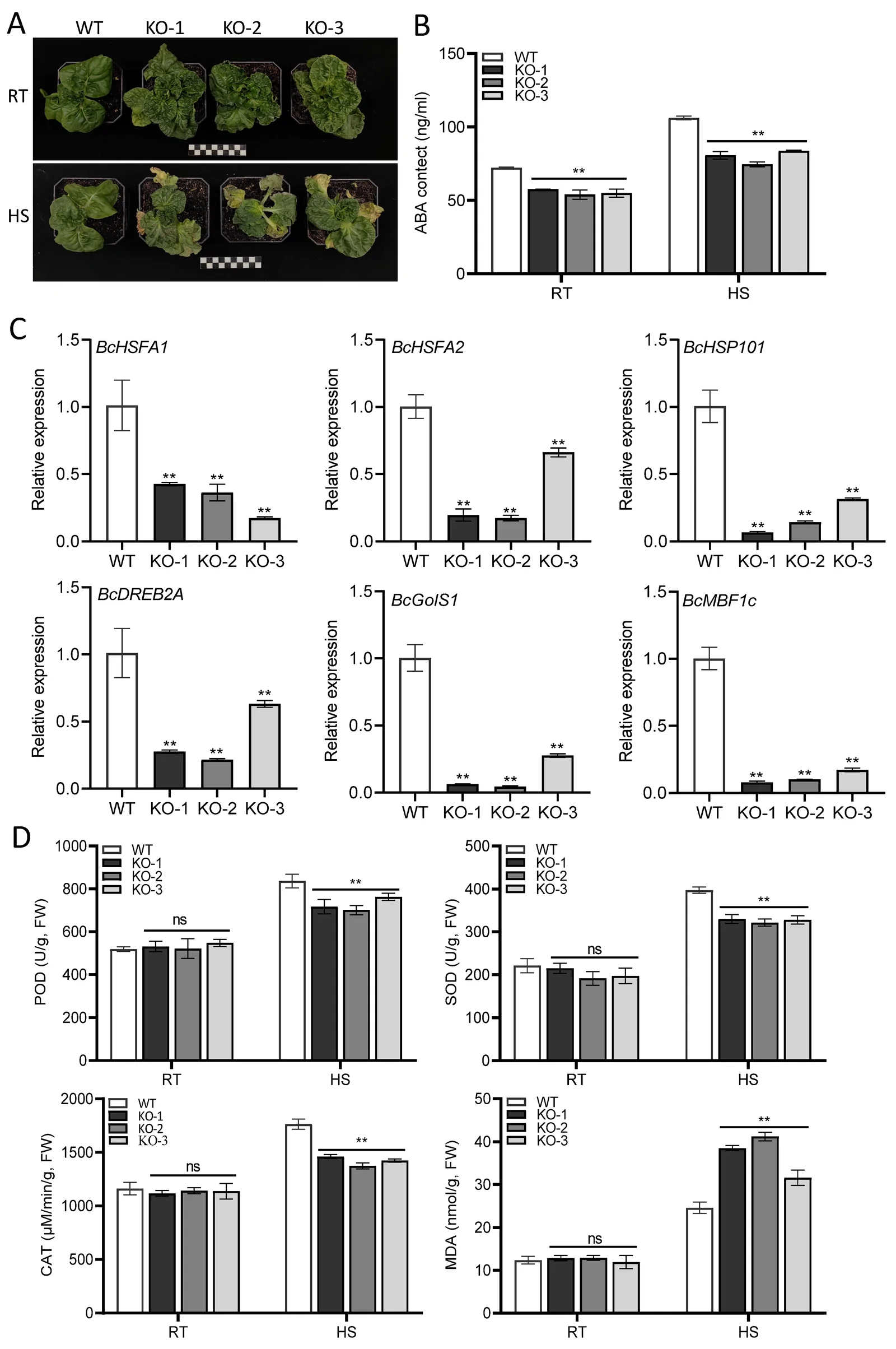
CRISPR/Cas12a editing of *BcNCED3* reduces heat tolerance in non-heading Chinese cabbage: (**A**) phenotypes of WT and *BcNCED3*-edited plants (KO-1, KO-2, and KO-3) at RT and HS; (**B**) endogenous ABA levels; (**C**) expression of heat-responsive genes *BcHSFA1*, *BcHSFA2*, *BcHSP101*, *BcDREB2A*, *BcGolS1*, and *BcMBF1c* after high-temperature treatment; (**D**) POD, SOD, and CAT activities and MDA content at RT and HS. Data are presented as mean ± SD (*n* = 3 biological replicates), (** *p *< 0.01, one-way ANOVA with Dunnett’s test for gene expression; two-way ANOVA with Tukey’s HSD for physiological indices, ns = non-significant).

After three days of cyclic 38 °C/25 °C thermal treatment, all three homozygous knockout (KO) lines developed severe foliar wilting and necrotic lesions, alongside accelerated vegetative growth relative to WT controls during stress incubation (Figure 5A). Although heat stimulation triggered measurable ABA biosynthesis in all genotypes, KO lines maintained significantly depleted endogenous ABA pools both prior to and post thermal challenge (Figure 5B). This genetic evidence confirms that functional ablation of *BcNCED3* severely attenuates stress-inducible ABA biosynthesis. Ablated ABA signalling renders knockout material refractory to thermal cues, disrupting the developmental switch from vegetative proliferation to stress acclimation. Under heat stress, all six heat-responsive marker genes exhibited markedly dampened transcription in KO lines relative to WT specimens (Figure 5C). While antioxidant enzymatic activity still increased in mutagenized material upon heat imposition, the magnitude of induction was significantly blunted, paired with substantially elevated MDA titres compared with wild-type tissue (Figure 5D). These physiological disparities reflect weakened ROS scavenging machinery and aggravated membrane lipid peroxidation in the absence of functional *BcNCED3*.

## 4. Discussion

Non-heading Chinese cabbage is a cool-season leafy vegetable with an optimal growth temperature range of 18–20 °C. Sustained ambient temperatures exceeding 25 °C severely inhibit vegetative growth, triggering premature petiole elongation, accelerating leaf chlorosis, increasing crude fiber deposition, and ultimately reducing both edible quality and marketable yield. Abscisic acid (ABA), a quintessential stress phytohormone, accumulates rapidly upon exposure to drought, salinity, and thermal stress, orchestrating a wide repertoire of stress-adaptive responses [56]. *NCED* enzymes catalyze the rate-limiting step of ABA biosynthesis; consequently, the transcriptional regulation of *NCED* genes governs cellular ABA biosynthetic capacity and directly shapes thermotolerance phenotypes in crop plants [16,31].

The *BcNCED3* coding sequence is contained within a single exon lacking intronic interruptions. This intronless genomic architecture circumvents post-transcriptional splicing, enabling rapid transcriptional and translational activation to sustain robust ABA production during environmental perturbations [57]. The deduced *BcNCED3* polypeptide harbors a conserved *RPE65* domain characteristic of the NCED protein superfamily, which endows intrinsic dioxygenase activity to catalyze the oxidative cleavage of *9-cis-epoxycarotenoids* during ABA biosynthesis [58]. Phylogenetic analysis revealed pronounced sequence conservation between BcNCED3 and NCED3 orthologs across *Brassicaceae* species, echoing evolutionary conservation patterns previously documented in peanut, radish, and barley [59,60]. In silico predictions and experimental subcellular localization assays collectively demonstrate chloroplast residency of *BcNCED3*, consistent with its core catalytic function in plastid-localized ABA biosynthesis. Quantitative RT-PCR analysis revealed robust and rapid transcriptional induction of *BcNCED3* under 40 °C heat stress, with markedly divergent transcript accumulation between heat-tolerant and heat-sensitive cultivars. This positive correlation between *BcNCED3* expression and thermotolerance mirrors the established function of *OsNCED1* in rice, where elevated *NCED1*-mediated ABA biosynthesis reinforces heat resilience in seedlings [61].

Exogenous phytohormone application represents a practical strategy to mitigate heat-induced cellular damage and reinforce basal thermotolerance [11]. In rice, pretreatment with 10 μM ABA alleviated heat-induced leaf wilting and chlorophyll degradation while transcriptionally upregulating antioxidant and heat shock gene repertoires [25]. Consistent with these findings in cereals, exogenous ABA application in non-heading Chinese cabbage significantly elevated *BcNCED3* transcript levels under thermal stress, augmented endogenous ABA concentrations, activated heat-responsive transcription factors (*BcHSP101*, *BcHSFA1*, and *BcHSFA2*), stimulated antioxidant enzyme activities, and suppressed MDA overaccumulation. These results indicate that exogenous ABA amplifies endogenous ABA biosynthesis while concurrently mobilizing heat-responsive transcriptional modules and antioxidant defense machinery, ultimately attenuating membrane lipid peroxidation [62,63,64]. Our observations align with prior studies attributing enhanced heat tolerance to ABA accumulation in mini Chinese cabbage and cucumber [65,66].

Notably, *BcNCED3*-overexpressing transgenic lines faithfully recapitulated the full spectrum of physiological, transcriptional, and phenotypic thermotolerance responses elicited by exogenous ABA treatment. Conversely, CRISPR/Cas12a-mediated knockout mutants displayed pronounced heat hypersensitivity. Although the core finding-that *NCED*-mediated ABA biosynthesis enhances thermotolerance through *HSF*/*HSP* and antioxidant pathways has been documented in several model and crop species [61,66], our study provides several distinct contributions specific to non-heading Chinese cabbage: (i) the first functional characterization of an *NCED* family member in this economically important leafy vegetable; (ii) direct evidence that *BcNCED3* expression level quantitatively correlates with thermotolerance across cultivars; and (iii) a complete gain- and loss-of-function genetic validation demonstrating the necessity and sufficiency of *BcNCED3* for heat tolerance. This dual genetic validation establishes *BcNCED3* as a positive regulator of thermotolerance in non-heading Chinese cabbage, operating through ABA-dependent *HSF*/*HSP* regulatory axes and antioxidant defense pathways [67].

Stable genetic transformation combined with precision genome editing provides a powerful toolkit for molecular dissection of plant gene function [54]. In this study, we employed *Agrobacterium rhizogenes*-mediated transformation alongside the LbCas12a-RRV multiplex editing platform to generate complementary overexpression and knockout germplasm. While CRISPR/Cas9 and conventional Cas12a systems have been successfully established in several Brassica species (e.g., *B. oleracea* and *B. napus*), their application in non-heading Chinese cabbage (*B. rapa* subsp. *chinensis*) remains limited, largely due to the recalcitrance of this subspecies to genetic transformation and tissue regeneration-a major bottleneck for functional genomics and molecular breeding. The LbCas12a-RRV system deployed in this work offers several practical advantages in the non-heading Chinese cabbage context: (i) the WUS2-IPT-PLT5 developmental regulator cassette carried in the vector backbone substantially enhances regeneration efficiency from transformed explants, with the Ruby visible reporter enabling early, non-destructive screening of positive transformants; (ii) Cas12a recognizes T-rich PAM sequences (TTTV), expanding targetable genomic space to AT-rich regulatory regions such as the ABRE- and W-box-enriched *BcNCED3* promoter; and (iii) Cas12a generates staggered DNA double-strand breaks with short overhangs, facilitating precise gene disruption through the *NHEJ* repair pathway—consistent with the 12–29 bp small deletions observed in this study [55]. Distinct from SpCas9 systems, LbCas12a requires only a single crRNA guide, recognizes AT-rich protospacer adjacent motifs, and produces cohesive-ended breaks conducive to precise allele modification [68,69]. This editing platform has been deployed with high efficiency across model organisms and diverse crop species [70,71,72,73,74,75]. Our functional validation confirms that LbCas12a-RRV drives consistent, high-efficiency mutagenesis in non-heading Chinese cabbage, furnishing a robust technical resource for functional genomic research within this crop and allied Brassicaceae vegetables. Critical gaps remain regarding upstream transcriptional regulators governing *BcNCED3* expression, as well as its nuanced regulatory roles linking ABA heat signalling to plant growth and developmental homeostasis [76], these lines of inquiry warrant dedicated follow-up characterisation.

## 5. Conclusions

This study demonstrates that chloroplast-localized *BcNCED3* positively regulates thermotolerance in non-heading Chinese cabbage through ABA biosynthesis modulation. Constitutive overexpression of *BcNCED3* significantly enhanced heat tolerance, whereas CRISPR/Cas12a-mediated knockout conferred marked heat hypersensitivity, establishing *BcNCED3* as a bona fide positive thermotolerance regulator. The validated high-efficiency LbCas12a-RRV genome editing platform and the functionally characterized *BcNCED3* gene together serve as valuable molecular tools and genetic resources for advancing heat-tolerance breeding in non-heading Chinese cabbage and allied *Brassica* vegetables.

## Figures and Tables

**Table 1:** Primer sequences used in this study.

Primer Name	Primer Sequence (5′–3′)	Usage
*BcNCED3*-F	ATGGCTTCTTTCACGGCGAATACGG	Gene cloning
*BcNCED3*-R	TTACACCTGCTTCGCCAAGTCACTA	Gene cloning
pRI101-*BcNCED3*-F	TCTTCACTGTTGATACAATGGCTTCTTTCACGG	Vector construction
pRI101-*BcNCED3*-R	GCCCTTGCTCACCATGGATCCCACCTGCTTCGCC	Vector construction
pER8-*BcNCED3*-F	AATCTCGATACACCAAATCGTCGATGGCTTCTTTCACGGCG	Vector construction
pER8-*BcNCED3*-R	GTAGCTCCGCTTCCTCGCACCTGCTTCGCCAAGTCACTA	Vector construction
q*BcActin7*-F	GTTGCTATCCAGGCTGTTCT	RT-qPCR (reference)
q*BcActin7*-R	AGCGTGAGGAAGAGCGTAAC	RT-qPCR (reference)
q*BcNCED3*-F	TTCTCACCGGACGGGACTAA	RT-qPCR
q*BcNCED3*-R	CCGGGAGCTTGAACACGAC	RT-qPCR

**Table 2:** Target site sequences for CRISPR/Cas12a editing.

Target Name	Target Sequence (5′–3′)
crRNA1-*BcNCED3*	CCCAAGCAGTCCTCAACCTCTCC
crRNA2-*BcNCED3*	GGGAAAACCGGTTTACCCAACTG

## Data Availability

The data presented in this study are available on request from the corresponding author. The data are not publicly available due to privacy restriction.

## Supplementary Materials

The following supporting information can be downloaded at https://www.mdpi.com/article/10.3390/plants15172613/s1. Figure S1: Analysis of cis-regulatory elements of *BcNCED3 *gene. Figure S2: Identification of *BcNCED3* overexpression plants: (A) GFP fluorescence photograph of regenerated seedling roots observed by LUYOR3415; (B) PCR detection of the *BcNCED3* gene in WT and *BcNCED3* overexpressing plants; (C) relative expression of *BcNCED3* in WT and *BcNCED3* overexpressing plants. Figure S3: Clustered ellipse correlogram showing pairwise correlations between *BcNCED3* gene expression, other tested genes and biochemical parameters. Positive correlations (*r* > 0, *p* ≤ 0.05) and negative correlations (*r* < 0, *p* ≤ 0.05) are distinguished by ellipse color. Figure S4: Mutation types of the *BcNCED3* ORF induced by CRISPR/Cas12a: (A) third-generation sequencing peak maps and alignment of wild-type (WT) and knockout (KO) plants; (B) mutation types at target site 1 in KO plants; (C) mutation types at target site 2 in KO plants.
